# CAM-A-dependent HBV core aggregation induces apoptosis through ANXA1

**DOI:** 10.1016/j.jhepr.2024.101134

**Published:** 2024-06-10

**Authors:** Valerio Taverniti, Laura Meiss-Heydmann, Cloé Gadenne, Hannah Vanrusselt, Dieudonné Buh Kum, Fabio Giannone, Patrick Pessaux, Catherine Schuster, Thomas F. Baumert, Yannick Debing, Eloi R. Verrier

**Affiliations:** 1 University of Strasbourg, Inserm, Institute for Translational Medicine and Liver Disease (ITM), UMR_S1110, Strasbourg, France; 2 Aligos Belgium BV, Leuven, Belgium; 3 Aligos Therapeutics, Inc., South San Francisco, CA, USA; 4 Institut Hospitalo-universitaire (IHU). Service d’hépato-gastroentérologie, Hôpitaux Universitaires de Strasbourg, Strasbourg, France; 5 Institut Universitaire de France, Paris, France

**Keywords:** Hepatitis B virus, Capsid assembly modulators, Apoptosis, *ANXA1*, Annexin A1, Lipocortin I

## Abstract

**Background & Aims:**

Chronic HBV infection is the leading cause of liver disease and of hepatocellular carcinoma. The improvement of antiviral therapy remains an unmet medical need. Capsid assembly modulators (CAMs) target the HBV core antigen (HBc) and inhibit HBV replication. Although CAM-A compounds are well-known inducers of aberrant viral capsid aggregates, their mechanisms of action in HBV-hepatocyte interactions are poorly understood. Recently, we demonstrated that CAM-A molecules lead to a sustained reduction of HBsAg in the serum of HBV replicating mice and induce HBc aggregation in the nucleus of HBc-expressing cells leading to cell death.

**Methods:**

The mechanism of action by which CAM-A compounds induce cell death was investigated using an HBV infection model, HBc-overexpressing HepG2-NTCP cells, primary human hepatocytes, and HBV replicating HepAD38 cells.

**Results:**

We first confirmed the decrease in HBsAg levels associated with CAM-A treatment and the induction of cell toxicity in HBV-infected differentiated HepaRG cells. Next, we showed that CAM-A-mediated nuclear aggregation of HBc was associated with cell death through the activation of apoptosis. Transcriptomic analysis was used to investigate the mechanism of action driving this phenotype. CAM-A-induced HBc nuclear aggregation led to the upregulation of ANXA1 expression, a documented driver of apoptosis. Finally, silencing of ANXA1 expression delayed cell death and apoptosis in CAM-A-treated cells, confirming its direct involvement in CAM-A-induced cell death.

**Conclusions:**

Our results unravel a previously undiscovered mechanism of action involving CAM-As and open the door to new therapeutic strategies involving CAM to achieve a functional cure in patients with chronic infections.

**Impact and implications:**

Chronic HBV infection is a global health threat. To date, no treatment achieves viral clearance in chronically infected patients. In this study, we characterized a new mechanism of action of an antiviral molecule targeting the assembly of the viral capsid (CAM). The study demonstrated that a CAM subtype, CAM-A-induced formation of aberrant structures from HBV core protein aggregates in the nucleus leading to cell death by ANXA1-driven apoptosis. Thus, CAM-A treatment may lead to the specific elimination of HBV-infected cells by apoptosis, paving the way to novel therapeutic strategies for viral cure.

## Highlights

• CAM-A treatment is associated with a decrease in secreted HBsAg levels and cell toxicity in HBV-infected dHepaRG cells.
• CAM-A treatment activates apoptosis in HBc-expressing cells through nuclear accumulation of HBc aggregates.
• Nuclear accumulation of HBc aggregates induces the upregulation of ANXA1 expression.
• ANXA1 drives apoptosis activation mediated by HBc aggregation and CAM-A treatment.

## Graphical abstract

**Figure:**
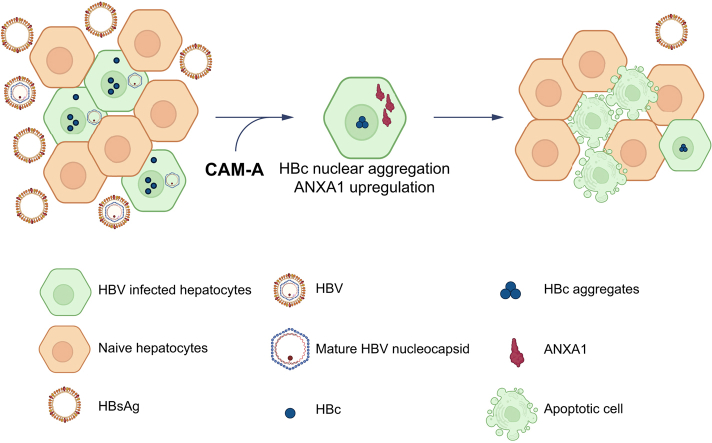

## Introduction

With approximately 300 million chronically infected patients worldwide, HBV is a leading cause of hepatocellular carcinoma (HCC), responsible for more than 800,000 deaths annually.^1^ Although an effective vaccine prevents infection, the current antiviral treatments based on nucleos(t)ide analogues (NUCs) only control viral replication in chronically infected patients, and viral cure is rarely observed.^2^ Thus, there is an urgent need for the development of novel therapeutic strategies against this major health threat. The current goal of antiviral therapy in development is to achieve an HBV ‘functional cure’, characterized by the loss of HBsAg in the serum of patients after 24 weeks off therapy.^2^ HBV is a small, enveloped, partially double-stranded DNA virus belonging to the *Hepadnaviridae* family, that almost exclusively infects human hepatocytes.^3^ Its replication cycle includes the formation of a covalently closed circular DNA (cccDNA), which is not targeted by the current therapies and serves as a template for the synthesis of viral RNAs.^4^ Among them, HBV pre-genomic RNA (pgRNA) allows both *de novo* genomic DNA synthesis upon reverse transcription by the viral polymerase and the production of the nucleocapsid protein, HBV core antigen (HBc).^5^ The HBV core protein contains 183 amino acids and exerts multiple key roles in HBV replication, cccDNA-mediated transcription to capsid formation and assembly.^6^ In this context, HBc has been intensively investigated as an antiviral target in the past decades. Notably, capsid assembly modulators (CAMs) directly interact with HBc, modulating capsid assembly and inhibiting HBV DNA production, and exhibiting promising results in clinical trials.^7^^,^^8^ CAMs have been divided into two classes according to their main mode of action.^6^ CAM E (formerly class II CAM) compounds lead to the formation of intact but empty capsids, CAM-A (formerly Class I CAM) molecules induce the formation of aberrant capsid structures and HBc aggregates by inhibiting the assembly of HBc multimers.^9^ Recently, we reported for the first time that treatment with RG7907, an heteroaryldihydropyrimidine (HAP) CAM-A leads to a sustained reduction of HBsAg in the serum of HBV replicating mice, shedding light on possible new antiviral mechanisms of action of CAM-A in HBV infection.^10^ Our preliminary investigations led to the hypothesis that CAM-A-induced HBc aggregates in the nucleus of HBc-expressing cells may trigger cellular apoptosis, named CCD (CAM-A-dependent cell death) leading to the elimination of infected cells and hepatocyte proliferation.^10^^,^^11^ These results were recently confirmed by an independent group in a HBV *in vivo* infection models.^12^ In this study, we developed several functional tools to further investigate and understand this finding, to characterize the mode of action by which CAM-A compounds induce cell death, and to identify host cell drivers of this new antiviral mechanism.

## Patients and methods

### Reagents and plasmids

Capsid assembly modulators CAM-A_h_ RG7907 and CAM-A_h_ GLS4 (HAP CAM-A), CAM-A_t_ ALG-005398, CAM-A_i_ ALG-005863, and CAM-A_t_ ALG-006162 (non-HAP CAM-A^11^), and compound B (reference CAM E^13^) were provided by Aligos Therapeutics (Leuven, Belgium), shipped as dried powder, and reconstituted in DMSO (Sigma-Aldrich, Merck, Germany). DNA transfection was performed using CalPhos Mammalian Transfection kit (Clontech, Takara Bio, USA) according to the manufacturer’s instructions. The following expression plasmids were obtained from VectorBuilder GmbH (Germany): HA-tagged HBV HBc wild type (HA:HBc WT, serotype ayw, genotype D) (VB210422-1136fgt); T33N mutant of HBc (HA:HBc T33N) (VB220105-1043neq); P25A mutant of HBc (HA:HBc P25A) (VB220105-1041sqy); I105F mutant of HBc (HA:HBc I105F) (VB220105-1046gga); HA control expression plasmid (VB220105-1047unq); lentiviral plasmid expressing shRNA targeting *ANXA1* mRNA (VB900130-7607sfz); and lentiviral plasmid expressing control shRNA (VB010000-0005mme). Vector sequences are available online (https://en.vectorbuilder.com/design/retrieve.html). Small guide (sg)RNA-encoding constructs were obtained from GenScript (SC1805, GenScript, USA). The ANXA1 gRNA sequence used was ATGCAAGGCAGCGACATCCG.

### Antibodies and primers

For details regarding the antibodies and primers used, please refer to the CTAT table.

### Cell lines and culture conditions

HepaRG cells^14^ were maintained in William’s medium (Sigma-Aldrich, Merck, Germany, W4128) supplemented with 10% foetal calf serum (PERBIO, France SH30066.03), penicillin/streptomycin 50 U/mL (Gibco, Thermo Fisher Scientific, USA 15140-148), GlutaMax 2 mM (Gibco, Thermo Fisher Scientific, USA, 35050-061), insulin bovine 5 μg/mL (Sigma, I9278), and 5 × 10^-5^ M hydrocortisone hemisuccinate (Sigma-Aldrich, Merck, Germany, 1319002) at 37 °C in humidified incubators at 5% CO2. To obtain differentiation of HepaRG, cells were maintained for 2 weeks in standard medium, then switched for at least 2 more weeks in medium supplemented with 1.8% DMSO (Sigma-Aldrich, Merck, Germany, D2650).

HepG2-NTCP cells have been described previously^15^ and were maintained in Dulbecco’s modified Eagle’s medium (DMEM, Gibco, Thermo Fisher Scientific, USA, 61965-059) supplemented with 10% foetal bovine serum (FBS, Dutscher, France, 500105k1k), 1X non-essential amino acids (NEAA, Gibco, Thermo Fisher Scientific, USA,11140-035), 50 μg/ml gentamicin (Gibco, Thermo Fisher Scientific, USA, 15710-049), and 250 μg/ml G418 (Invivogen, Thermo Fisher Scientific, USA, ant-gn-5). Primary human hepatocytes (PHH) were obtained from liver tissue from patients undergoing liver resection for liver metastasis at the Strasbourg University Hospitals with informed consent. Protocols were approved by the local Ethics Committee of the Strasbourg University Hospitals (CPP) and the Ministry of Higher Education and Research of France (DC 2016 2616). PHHs were isolated and cultured as described^16^ and maintained in William’s E medium (Sigma, W4128) supplemented with 35 mg/ml BSA, 5 ml Insulin Transferrin Selenium (Gibco, Thermo Fisher Scientific, USA, 41400045), 10^-7^ M dexamethasone (Sigma, D-2915), 1X NEAA (Gibco, Thermo Fisher Scientific, USA,11140-035), 50 μg/ml penicillin-streptomycin (Gibco, Thermo Fisher Scientific, USA, 15140-122), and 5 ml Glutamax 1% (Gibco, Thermo Fisher Scientific, USA, 35050-038). The HepAD38 cell line is an inducible (TET-OFF) human hepatoblastoma cell line harbouring an integrated tetracycline-responsive 1.2-fold HBV genome (serotype ayw, genotype D)^17^ and was propagated in absence of doxycycline in DMEM (Gibco, Thermo Fisher Scientific, USA, 42430-082) supplemented with 10% FBS, 5 μg/ml Insulin from bovine pancreas (Sigma-Aldrich, Merck, Germany, I6634), 25 μg/ml hydrocortisone (Sigma-Aldrich, Merck, Germany, H0396-100MG), 500 μg/ml G418 (Invivogen, Thermo Fisher Scientific, USA, ant-gn-5), and 50 μg/ml gentamicin (Gibco, Thermo Fisher Scientific, USA, 15710-049). Cells were incubated at 37 °C with 5% CO_2_.

### HBV production and infection

The production of recombinant HBV (ayw) infectious particles from HepAD38 cells has been described.[18], [19], [20] HepaRG cells were infected with HBV at a multiplicity of infection (MOI) of 500 viral genome equivalents per cell (vge/cell) in presence of 4% PEG as previously described.[18], [19], [20], [21] Infected cells were treated with the different CAMs at 10 μM, and the medium was refreshed in presence of the compounds every 5 days. HBV infection was quantified as described previously.^15^^,^[18], [19], [20] (See also CTAT table for the qPCR primers and probes).

### Lentiviral transduction

Individual HA:HBc-expression or shRNA-encoding or sgRNA-encoding lentiviral particles were produced in HEK 293T cells by cotransfection of human immunodeficiency virus (HIV) gag-pol, the vesicular stomatitis virus glycoprotein (VSV-G), and the corresponding pLenti plasmids using the CalPhos Mammalian Transfection kit as described previously.^19^ Three days after transfection, supernatants were collected and clarified using 0.45 μm pore filters. HepG2-NTCP were transduced with shRNA-encoding lentivirus and selected with puromycin 0.9 μg/ml. Alternatively, PHH were transduced with individual HA:HBc-containing lentivirus for 3 days prior to CAM treatment.

### Cytotoxicity assay

The cell supernatant was collected at the indicated time points and the lactate dehydrogenase (LDH) activity was measured using the LDH-Glo Cytotoxicity Assay (Promega, USA, J2380) following the manufacturer’s instructions.

### Cell viability and apoptosis assays

Cells were seeded in 96-well plate at a density of 2.3 × 10^4^ cells/well in 100 μl of culture medium and allowed to adhere for 24 h. Then, to treat HA:HBc expressing HepG2-NTCP cells, culture medium was replaced with PHH medium supplemented with CAM compounds at 1 μM or DMSO for the non-treated control. All tested conditions contained 2% DMSO final concentration. To treat HepAD38 cells, the culture medium was replaced with production medium: DMEM (Gibco, Thermo Fisher Scientific, USA, 42430-082) supplemented with 2% FBS, 5 μg/ml Insulin from bovine pancreas (Sigma-Aldrich, Merck, Germany, I6634), 25 μg/ml hydrocortisone (Sigma, H0396-100MG), 50 μg/ml gentamycin (Gibco, Thermo Fisher Scientific, USA, 15710-049) supplemented with the indicated CAM compounds in 2% DMSO final concentration. Medium and compounds were refreshed every 6-7 days. To treat PHH, after 3 days of lentiviral transduction, the culture medium was refreshed in presence of the indicated CAM compounds in 2% DMSO final concentration. Following treatment, cellular viability was determined using the resazurin-based PrestoBlue reagent (Invitrogen A13262). Briefly, 10 μl PrestoBlue solution was added into each well; plates were then incubated for 1 h prior to measuring the absorbance at 570 nm. The number of cells in each well was quantified by DAPI staining and counted using a Celigo Image Cytometer (Revvity, USA). To quantify apoptosis, cells were incubated with the Cell Event Caspase-3/7 Green Detection Reagent (Invitrogen, Thermo Fisher Scientific, USA, C10723) following the manufacturer’s instructions. Briefly, the assay relies on a Caspase-specific peptide linked to a fluorochrome. Activated caspases proteolytically cleave the peptide and release the fluorochrome that in turn binds to the DNA emitting green fluorescence. Microphotographs and quantification were obtained using a Celigo Image Cytometer.

### Quantification of HBsAg-expressing cells by flow cytometry

CAM-treated HepaRG cells were collected and crosslinked in 500 μl of 2% paraformaldeide for 15 min on ice. Then, cells were permeabilized in 200 μl permeabilization buffer (saponin 0.1%, FBS1%, EDTA 5mM, PBS 1X), for 16 h at 4 °C. Cells were then centrifuged at 1500 rpm for 5 min, resuspended in 50 μl of permeabilization buffer containing primary anti-HBsAg antibody (Bio-Techne, USA, NB100-64554) diluted 1/2000 and incubated for 30 min in ice. Then, 150 μl of permeabilization buffer were added to the cells and centrifuged. Cells were washed, resuspended in 50 μl of secondary anti-mouse AF488 diluted 1/2000 in permeabilization buffer, and incubated for 30 min in ice. Finally, cells were washed two times in PBS 1X, EDTA 5 mM, resuspended in 200 of PBS1X EDTA 5 mM and analysed by flow cytometry using the CytoFLEX instrument (Beckman Coulter, USA).

### Analysis of secreted HBsAg

From cell culture supernatants, secreted HBs antigens was quantified by ELISA, using a chemiluminescence immunoassay kit (AutoBio, China) according to manufacturer's instructions.

### Quantification of secreted HBV DNA

HepAD38 cells were washed with Dulbecco's phosphate-buffered saline (D-PBS), trypsinized, collected in culture medium and diluted at 2.5 × 10^5^ cells/ml. Next, 100 μl of cell suspension was seeded in 96-well plates corresponding to 25,000 cells per well and incubated at 37 °C with 5% CO_2_. 24 h later, culture medium was replaced by production medium supplemented with 2% DMSO or 1 μM of CAM compounds. Plates were incubated at 37 °C and 5% CO_2_ for the indicated time.

Alternatively, HBV-infected dHepaRG were treated with the indicated CAM compounds. CAM-containing medium was refreshed every 5 days.

The cell supernatant was collected, and the HBV DNA was extracted using QIAamp DNA Mini kit (Qiagen, Netherlands, 51306) following the manufacturer instructions. HBV DNA was quantified by qPCR as described^18^ using Bio-Rad (USA) CFX96 following the manufacturer’s instructions. The primers used for HBV DNA quantification (BC1 and PGP) are listed in the CTAT table.

### HBc immunofluorescence staining

Cells were fixed with 2% paraformaldehyde (PFA). HA:HBc was immunodetected using a specific rabbit polyclonal anti-HA antibody (ab9110, Abcam, UK) and Alexa Fluor 647-labelled secondary antibody targeting rabbit IgGs (Jackson ImmunoResearch, UK). Untagged HBc was immunodetected using a specific mouse monoclonal antibody (ab8637 Abcam, UK) and Alexa Fluor 488-labelled secondary antibody targeting mouse IgGs (Jackson ImmunoResearch). Cell nuclei were stained with DAPI. Fluorescent imaging was performed using a confocal microscope Zeiss LSM 800 AiryScan (Carl Zeiss, Germany).

### RNA-sequencing

Next-generation sequencing (and data analysis) was performed by the Biomedical Sequencing Facility at CeMM Research Center for Molecular Medicine of the Austrian Academy of Sciences (Vienna, Austria).

#### NGS library preparation

RNA-seq libraries were prepared with the NEB-Next® Ultra™ II Directional RNA sample preparation kit (New England Biolabs, Inc., Ipswich, MA, USA). NGS library concentrations were quantified with the Qubit 2.0 Fluorometric Quantitation system (Life Technologies, Carlsbad, CA, USA) and the size distribution was assessed using the 2100 Bioanalyzer instrument (Agilent, Santa Clara, CA, USA).

#### Next-generation sequencing and raw data acquisition

Expression profiling libraries were sequenced on a HiSeq 3000 instrument (Illumina, San Diego, CA, USA) following a 50-base-pair, single-end recipe. Raw data acquisition (HiSeq Control Software, HCS, HD 3.4.0.38) and base calling (Real-Time Analysis Software, RTA, 2.7.7) was performed on-instrument, whereas the subsequent raw data processing off the instruments involved two custom programs based on Picard tools (v.2.19.2). In a first step, base calls were converted into lane-specific, multiplexed, unaligned BAM files suitable for long-term archival (IlluminaBasecallsToMultiplexSam, 2.19.2-CeMM). In a second step, archive BAM files were demultiplexed into sample-specific, unaligned BAM files (IlluminaSamDemux, 2.19.2-CeMM).

#### Transcriptome analysis

NGS reads were mapped to the Genome Reference Consortium GRCh38 assembly via ‘Spliced Transcripts Alignment to a Reference’ (STAR, 2.7.9a) utilizing the ‘basic’ ensembl transcript annotation from version e100 (April 2020) as the reference transcriptome. Since the hg38 assembly flavour of the UCSC Genome Browser was preferred for downstream data processing with Bioconductor packages for entirely technical reasons, ensembl transcript annotation had to be adjusted to UCSC Genome Browser sequence region names. STAR was run with options recommended by the ENCODE project. NGS read alignments overlapping ensembl transcript features were counted with the Bioconductor (v.3.14) Genomic Alignments (v.1.30.0) package. Transcript-level counts were aggregated to gene-level counts and the Bioconductor DESeq2 (v.1.34.0) package was used to test for differential expression based on a model using the negative binomial distribution. The expression of 20 genes related to apoptosis according to HALLMARK (Human MSigDB Collections) and significantly upregulated upon CAM-A treatment were determined using the Z-score transformation.

### Quantification of gene expression by RT-qPCR

Total RNA was extracted using ReliaPrep RNA Miniprep Systems (Promega, USA) and reverse-transcribed into complementary DNA (cDNA) using the Maxima First Strand cDNA Synthesis Kit (Thermo Fisher Scientific, USA) according to the manufacturer’s instructions. Gene expression was then quantified by quantitative PCR using an Applied Biosystems instrument. Primers and TaqMan® probes for *ANXA1* and *GAPDH* mRNA quantification were obtained from ThermoFisher (TaqMan Gene Expression Assay; Applied Biosystems, Thermo Fisher Scientific, USA). References are listed in the CTAT table. Primers and TaqMan® probe for the quantification of HBV precure RNA and pgRNA (pc/pgRNA) were the following: pc/pgRNA Fw primer: 5′-GGTCCCCTAGAAGAAGAACTCCCT-3′; pc/pgRNA Re primer: 5′-CATTGAGATTCCCGAGATTGAGAT-3′; and pc/pgRNA probe: 5′-[6FAM]-TCTCAATCGCCGCGTCGCAGA-[BHQ1]-3′. All values were normalized to *GAPDH* expression.

### Detection of protein expression by Western blot

The expression of HA:HBc, ANXA1, β-tubulin, and β-actin proteins were assessed by Western blot as described previously^15^^,^^20^^,^^22^ using a monoclonal HRP-conjugated anti-HA antibody (Roche 11867423001), an anti-ANXA1 antibody (Abcam, UK, Ab214486), a monoclonal anti-β-actin antibody (AbCAM-AB8226), and an anti-β-tubulin antibody (Gentex, USA, GTX101279), respectively. Corresponding HRP-conjugated secondary antibodies were obtained from Jackson ImmunoResearch (UK).

### Data analyses

The number of independent experiments per assay is indicated in the figure legends. Quantitative data are expressed as means + SD relative to the control condition set at 100% except otherwise stated in the figure legends. For assays with more than four biological replicates, statistical analyses were performed when appropriate using a two-tailed Mann-Whitney U test. For Western blots and immunofluorescence assay images, one representative experiment is presented. All the graphics and analyses were performed on GraphPad Prism v.9.

## Results

### CAM-A treatment induces a decrease in secreted HBsAg levels and cellular toxicity in HBV-infected dHepaRG cells

**Fig. 1:**
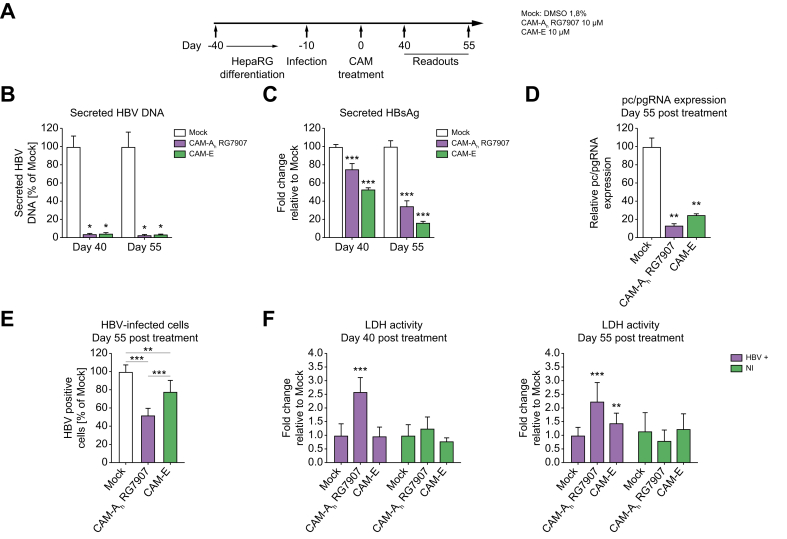
CAM-As-induce apoptosis in HBV infected dHepaRG. (A) HepaRG cells were differentiated, infected with HBV, and treated with the indicated compounds after 10 days of infection. (B-D) The efficiency of CAM treatment was assessed after 40- and 55-days post-treatment by measuring: the amount of secreted HBV DNA (B), pc/pgRNA expression (C), and HBsAg secretion (D). (E) The percentage of HBV-infected dHepaRG was measured by flow cytometry by sorting HBsAg positive cells. (F) Cytotoxicity was quantified by measuring LDH activity in the cell supernatant of treated cells. Values were normalized to mock treated cells set by default at 1. Data are expressed as means of two independent experiments. Levels of significance: ∗*p* <0.05, ∗∗*p* <0.01, and ∗∗∗*p* <0.001 (two-tailed Mann–Whitney U test).

First, we validated our previous findings in an HBV-infection assay. We assessed the effect of CAM-A treatment on cell viability in HBV-infected differentiated HepaRG cells, a highly relevant HBV-infection system allowing long-term infection periods Two independent HBV infection assays HBV (genotype D, serotype ayw) at 500 vge/cell were performed with comparable infection rates after 10 days (Fig. S1A). At Day 10 post-infection, we started treatment with CAM-A_h_ RG7907, and CAM-E at 10 μM (Fig. 1A). We observed in a proof-of-principle assay that although the viral load in the cell supernatant was reduced upon short-term CAM treatment (20 days) because of their primary antiviral activity, the level of secreted HBsAg was not affected (Fig. S1B-C). Moreover, we observed decreased levels of secreted HBsAg as well HBV RNA production after longer treatment with CAM-A (Fig. 1B-D), confirming our and other observations[10], [11], [12] as well as previous results suggesting multiple antiviral mechanisms of action in HBV-infected cells upon long-term treatments with CAMs.^23^ Interestingly, secreted HBsAg and intracellular RNA levels were also affected by prolonged CAM-E treatment (Fig. 1B-D), which was in line with previous results suggesting that CAM-E may affect early steps of the viral cycle, before antigen production.^24^ Surprisingly, this inhibition of secreted HBsAg levels were associated with a more pronounced decrease in the total number of HBV infected cells in CAM-Ah RG7907-treated cells (Fig. 1E) as well as nuclear aggregation of HBc (Fig. S2) compared with CAM-E treated cells, suggesting two different antiviral modes of action explaining these secondary antiviral mechanisms. Interestingly, we also observed an increased cytotoxicity after long treatment of HBV-infected cells with CAM-A_h_ RG7907 (Fig. 1F). This result is in line with our previous results on CAM-A dependent CCD. Short term treatment did not alter cell viability (Fig. S2B) indicating that the CCD is a very slow process dependent on the gradual accumulation of nuclear HBc aggregates, as suggested by a recent study.^12^ In contrast, CAM-A_h_ RG7907 treatment did not induce CCD in non-infected cells (Fig. 1F). Altogether, these data indicated that CAM-A_h_ RG7907 treatment induces cell death of HBV-infected dHepaRG likely through the accumulation of nuclear HBc aggregation. However, given that this process is slow and requires a high level of HBc, we moved to alternative *in vitro* system to understand mechanism of action involved in CCD.

### CAM-A-mediated HBc aggregation is associated with CCD

**Fig. 2:**
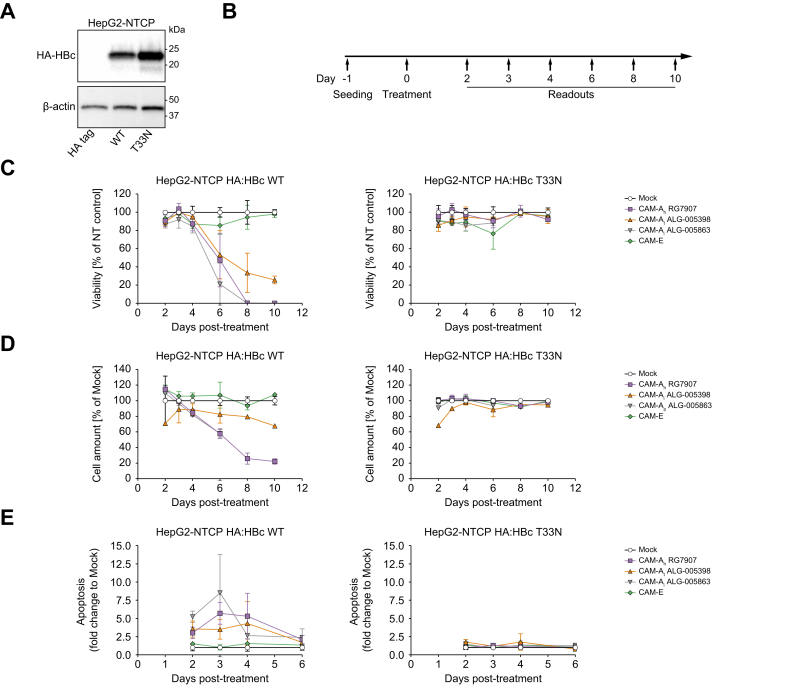
CAM-As induce apoptosis in HBc-overexpressing cells. (A) Western blotting of HA:HBc WT, or HA:HBc T33N mutant-overexpressing HepG2-NTCP cell lysates probed for HA. (B-E) HA:HBc WT, or HA:HBc T33N mutant-overexpressing HepG2-NTCP cell were either mock-treated with 2% DMSO or treated with either CAM-A_h_ RG7907 or CAM-A_t_ ALG-005398 or CAM-A_i_ ALG-005863 or CAM-E (final concentration 1 μM) for the indicated time (B) and assessed for cell viability by PrestoBlue (C), total cell amount by DAPI staining (D), and apoptosis determined by a caspase 3/7 reporter assay (E). Cell viability and cell amount values relative to CAM treatments were normalized to mock treatment for each time point set by default at 100%. Apoptosis values relative to CAM treatments were normalized to mock treatment set by default at 1 for each time point. Data are expressed as means of one to three independent experiments.

To investigate the antiviral mechanism of action specific to CAM-A compounds, we produced HepG2-NTCP cell lines that stably express the full length (183 aa) HBc protein (genotype D, serotype ayw) fused to a HA-tag at its N-terminus (Fig. 2A). In addition, we generated cell lines expressing several CAM-resistant HBc mutants, notably T33N, I105F, and P25A (Fig. 2A, Fig. S3A).^25^ These three mutations do not abrogate the ability of HBc to form mature capsid structures and are found in nature or as a CAM treatment-emergent mutation.^25^^,^^26^ Next, we treated the corresponding cell lines with CAM-A_h_ RG7907 or CAM-A_h_ GLS4 (belonging to the HAP series), or recently described non-HAP CAM-As, notably CAM-A_t_ ALG005398, CAM-A_i_ ALG005863 and CAM-A_t_ ALG006162,^11^ or CAM-E (compound B) as indicated in (Fig. 2B, Fig. S4). Treatment with CAM-A compounds resulted in a strong decrease in the viability and the number of cells expressing HBc WT as compared with mock treated cells while the viability and the number of cells treated with CAM-E compound was not affected (Fig. 2C and D left panels, Fig. S3B, Fig. S4A and B). We also observed that CAM-A_t_ ALG005398 (green line) exhibited a delayed effect on cell viability compared with the CAM-A_h_ or CAM-A_i_ (respectively pink and grey lines). This observation is in line with our recently published data.^11^

Interestingly, CAM-A treatment did not alter the viability of cells expressing the HBc T33N mutant (Fig. 2C and D, right panels) consistent with the inability of CAM compounds to bind the HBc T33N hydrophobic pocket.^25^ Surprisingly, HBc with I105F or P25A mutations could be targeted by CAM-A molecules, although their ability to induce cell death was less efficient. While all CAM-As affected the viability of P25A HBc mutant expressing cells, only CAM-A_h_ RG7907, but not CAM-A_t_ ALG-005398 and CAM-A_i_ ALG-005863, altered the viability of I105F HBc mutant expressing cells (Fig. S4B). Taken together, our results demonstrate that CAM-A compounds induced cell death in a HBc-dependent manner.

### Nuclear HBc aggregation induced apoptosis in a CAM-A-dependent manner

**Fig. 3:**
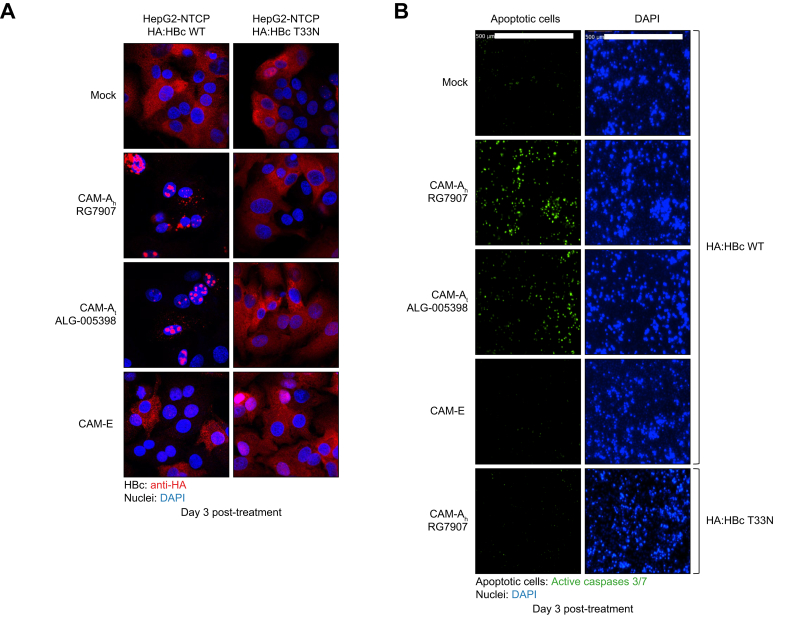
Apoptosis is associated with HBc aggregation upon CAM treatment. (A) Immunofluorescent staining for HBc (red) and DAPI-stained nuclei (blue) in HA:HBc WT, or HA:HBc T33N mutant-overexpressing HepG2-NTCP cells either mock-treated with DMSO or treated with either CAM-A_h_ RG7907, CAM-A_t_ ALG-005398, or CAM-E for 3 days. (B) From similar samples, the monitoring of apoptosis was performed using a caspase 3/7 reporter assay.

To determine the trigger for CCD, we first evaluated the distribution of HBc following CAM treatment using an immunofluorescence assay. In mock treated cells expressing HBc WT, HBc was equally distributed both in the cytoplasm and nucleus while in CAM-E treated cells HBc was mainly cytoplasmic (Fig. 3A and Fig. S5A). Importantly, the presence of the HA:tag did not influence the cellular localization of HBc as compared with the untagged protein (Fig. S5B). Thus, CAM-A treatment induced the nuclear accumulation of HBc aggregates and the loss of cytoplasmic HBc, confirming our previous observations (Fig. 3A).^10^^,^^11^ As expected, CAM treatment did not influence the HBc distribution in cells expressing the HBc T33N mutant (Fig. 3A). We then studied whether CAM-A treatment could induce apoptosis. Interestingly, in the CAM-A-treated cells we observed an increase over time in the levels of apoptosis relative to the mock treated cells while the CAM-E compound did not affect apoptosis levels (Fig. 2E left panel, Fig. 3B, Fig. S3C, Fig. S4C). As expected, CAM-A compounds did not activate apoptosis in cells expressing the HBc T33N mutant (Fig. 2E, right panel; Fig. 3B, lower microphotographs) confirming the previous observations on cell viability. Collectively, these results demonstrate that CAM-A compounds induced nuclear HBc aggregation associated with the activation of apoptosis.

### HBc aggregation induced apoptosis in HBc overexpressing PHH and HBV replicating HepAD38

**Fig. 4:**
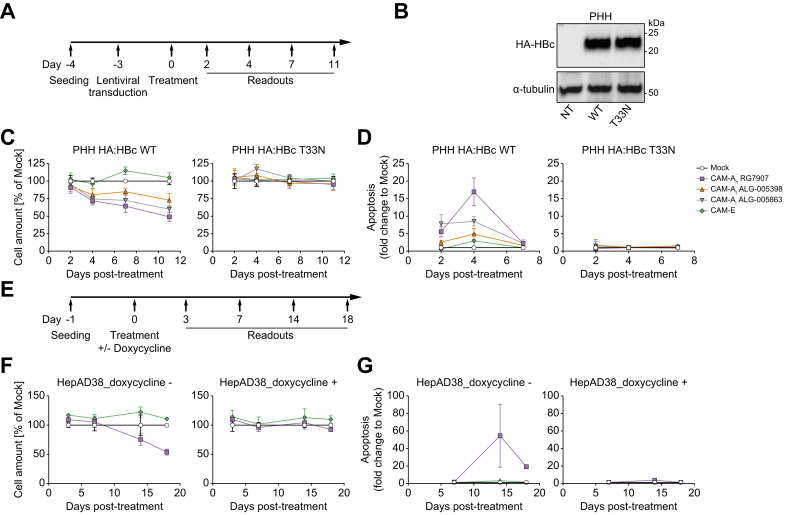
CAM-As induces apoptosis in HBc-overexpressing PHH and in HBV replicating HepAD38. (A-B) HA:HBc WT, or HA:HBc T33N mutant-overexpressing PHH were either mock-treated with 2% DMSO or treated with either CAM-A_h_ RG7907, CAM-A_t_ ALG-005398, CAM-A_i_ ALG-005863, or CAM-E (final concentration 1 μM) for the indicated times. Western blotting of HA:HBc WT, or HA:HBc T33N mutant-overexpressing PHH lysates probed for HA (B). PHH total cell amount was assessed by DAPI staining (C), and apoptosis levels were assessed by a caspase 3/7 reporter assay (D). (E) HepAD38 cells were either mock-treated with 2% DMSO or treated with either CAM-A_h_ RG7907 or CAM-E (final concentration 1 μM) in the absence or in presence of doxycycline to inhibit HBV replication for the indicated times. (F) The efficiency of CAM treatment was assessed by measuring the amount of secreted HBV DNA. (G) Treated cells were assessed for total cell amount by DAPI staining and apoptosis by a caspase 3/7 reporter assay. Cell levels and HBV-secreted DNA values relative to CAM treatments were normalized to mock treatment set by default at 100%. Apoptosis values relative to CAM treatments were normalized to mock treatment set by default at 1. PHH data are expressed as the means from four biological replicates. HepAD38 data are expressed as means from two independent experiments.

To investigate whether HBc aggregation-dependent apoptosis also occurs in hepatocytes, the natural host of HBV, we transduced PHH with lentiviral vectors encoding HA:HBc WT and T33N mutant and cultured them for 3 days to enable HA:HBc protein expression (Fig. 4A and B). Next, we treated transduced PHH with mock or CAM compounds as indicated in Fig. 4B. In PHH expressing HBc WT, CAM-A treatment reduced the number of cells as compared with the mock treatment, whereas exposure to the CAM-E compound did not affect the number of cells (Fig. 4C left panel). Moreover, CAM-A compounds increased the levels of apoptosis but CAM-E did not (Fig. 4D, left panel). As expected, CAM-A treatment did not induce cell death in PHH expressing the HBc T33N mutant (Fig. 4C and D, right panels). This observation confirms the relevance of HBc aggregation associated with cell death in well-differentiated human hepatocytes.

To further investigate the effects of CAM-A treatment on cell viability in presence HBV antigen production and replication, we treated HBV-producing HepAD38 cells with CAM-A_h_ RG7907, CAM-E, or mock (Fig. 4E). Treatment with both CAM-A_h_ RG7907 and CAM-E inhibited secretion of mature HBV DNA viral particles validating the antiviral effect of CAMs (Fig. S6). Although mock or CAM-E treatment did not affect cell viability and apoptosis, CAM-A treatment resulted in a strong reduction of cell viability and activation of apoptosis (Fig. 4F-G, left panels). Conversely, CAM-A treatment did not affect the viability of HepAD38 cells cultivated in presence of doxycycline which inhibits pgRNA transcription from the TET-OFF promoter (Fig. 4F-G, right panels), although viral replication was not 100% inhibited (Fig. S6). Taken together, our results confirm that CAM-A treatment induced apoptosis in HBV-replicating cells.

### HBc aggregation induced the upregulation of *ANXA1*

**Fig. 5:**
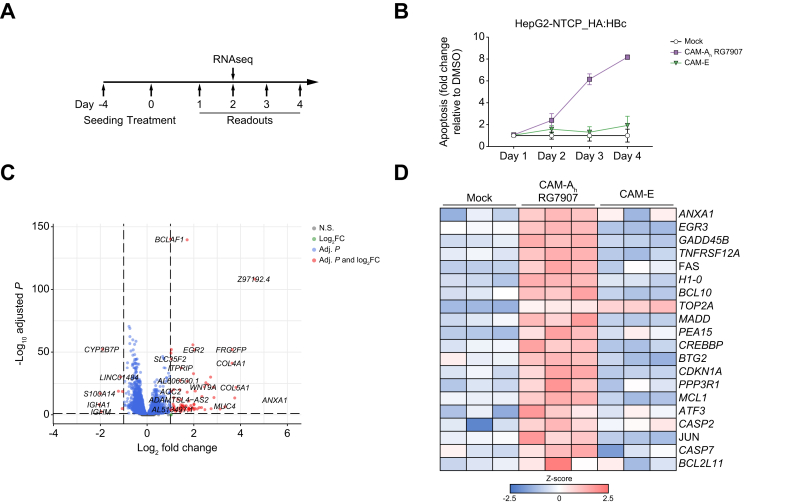
CAM-A_h_ RG7907 dependent HBc aggregation induced the specific upregulation of apoptosis-related genes. (A) Experimental setup. (B) HA:HBc WT-overexpressing HepG2-NTCP cell were either mock-treated with 2% DMSO or treated with either CAM-A_h_ RG7907 or CAM-E (final concentration 1 μM) for the indicated times and apoptosis levels were assessed by a caspase 3/7 reporter assay. (C) Volcano plot of genes exhibiting significantly modulated expression upon CAM-A_h_ RG7907 treatment versus mock-treated cells 2 days post-treatment analysed by RNAseq. (D) Differential gene expression of apoptosis-related genes in CAM-A_h_ RG7907, or mock or CAM-E treated HA:HBc overexpressing HepG2-NTCP cells.

Next, we aimed to identify the cellular drivers of apoptosis in CAM-A-treated cells. Thus, we treated HBc-expressing HepG2-NTCP cells with CAM-A_h_ RG7907 or CAM-E for 4 days (Fig. 5A). Apoptosis started on Day 2 with a peak at Day 4 (Fig. 5B). To identify genes driving apoptosis, we performed RNAseq analysis at Day 2 post-treatment, when apoptosis was barely detectable. CAM-A_h_ RG7907 treatment induced significant deregulation of several host genes (Fig. 5C). Notably, several genes playing a key role in apoptosis were upregulated upon CAM-A_h_ RG7907 treatment in comparison with the mock treated or CAM-E treated cells (Fig. 5D), including *ANXA1*, which encodes for annexin A1 a member of the annexin family of proteins.^27^ and EGR3.

**Fig. 6:**
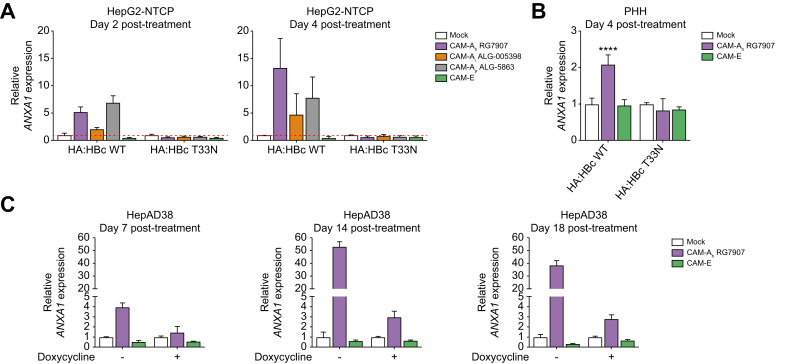
ANXA1 is upregulated upon CAM-A treatment. (A). *ANXA1* expression levels measured by RT-qPCR after treatment with either DMSO 2%, CAM-As, or CAM-E in HepG2-NTCP HA:HBc WT or T33N mutant at Day 2 (left panel) or 4 days (right panel) post-treatment. (B). *ANXA1* expression in PHH expressing HA:HBc WT or T33N mutant. (C) Time-dependent *ANXA1* expression in HepAD38 in the presence or absence of doxycycline. *ANXA1* expression was normalized to *GAPDH* expression and expressed as fold change relative to mock-treated cells set at 1. HepG2-NTCP and HepAD38 data are proof-of-principle assays from one validation experiment performed with three biological replicates. PHH data are expressed as means from two independent experiments. Levels of significance: ∗*p* <0.05, ∗∗*p* <0.01, and ∗∗∗*p* <0.001 (two-tailed Mann–Whitney U test).

We initially excluded EGR3 as bona-fide apoptosis driver as its expression is not affected in HBc expressing PHH following CAM-A treatment (data not shown). Hence, we focused on ANXA1 because this factor is known for its role in the anti-inflammatory response and apoptosis.[28], [29], [30], [31] We first validated the upregulation of *ANXA1* in CAM-treated HBc-expressing cells in a proof-of-principle assay involving three independent models. All CAM-As available in this study induced a significant upregulation of *ANXA1* mRNA levels relative to mock treatment in HepG2-NTCP expressing HA:HBc WT (Fig. 6A). As expected, CAM-E treatment did not affect *ANXA1* expression (Fig. 6A). Furthermore, we observed that *ANXA1* expression increased over time (Fig. 6A, compare left and right graphs). Interestingly, CAM-A_t_ ALG-005398 presented the less pronounced induction of ANXA1, associated with a lower induction of apoptosis and delayed CCD. In PHHs expressing HBc WT, CAM-A_h_ RG7907 also induced the upregulation of *ANXA1* (Fig. 6B) indicating that CAM-A dependent *ANXA1* upregulation was not attributable to dysregulated pathways specific to cancer cell lines. In addition, *ANXA1* expression levels did not change after CAM-A treatment in both systems expressing the HBc T33N mutant (Fig. 6A and B) indicating that *ANXA1* upregulation was a direct effect of HBc aggregation. Finally, this observation was confirmed in HBV-replicating HepAD38 cells treated with CAM-A_h_ RG7907 (Fig. 6C); CAM-E treatment did not affect *ANXA1* expression. The weak upregulation of *ANXA1* in presence of doxycycline in HepAD38 cells (Fig. 6C, Days 14 and 18 post treatment, doxycycline and related graphs) is likely a result of the residual viral replication previously observed (Fig. S6). Altogether, our results demonstrate CAM-A-dependent deregulation of apoptosis-related gene expression, including *ANXA1*.

### ANXA1 acted as a driver of apoptosis following HBc aggregation

**Fig. 7:**
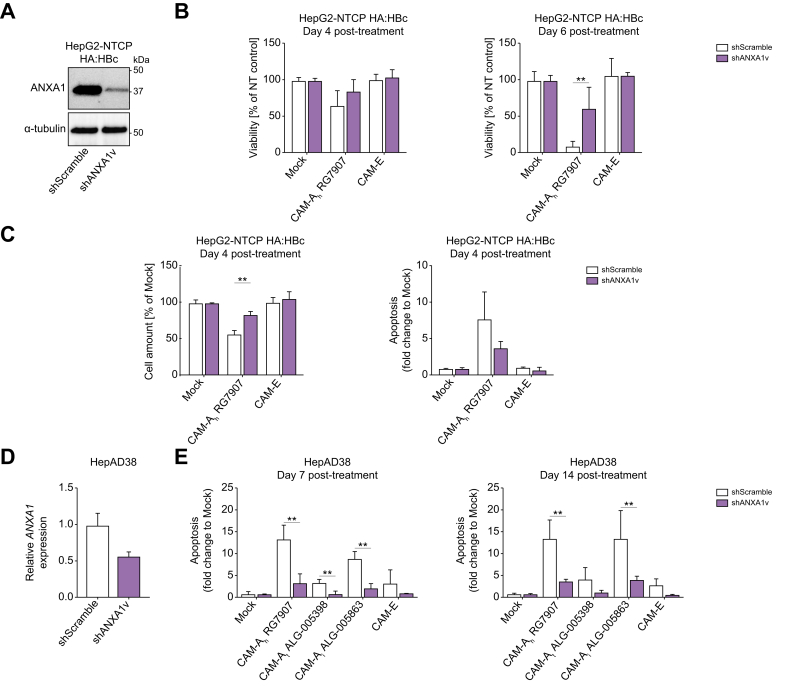
Loss of ANXA1 expression delays CAM-A-dependent cell death. (A) ANXA1 protein levels in HepG2-NTCP HA:HBc expressing non-targeting shRNA or shRNA targeting *ANXA1* mRNA measured by Western blot. (B-C) Cells were either mock-treated or treated with either CAM-A or CAM-E and cell viability was assessed by PrestoBlue assay (B). Total cell amount was assessed by DAPI staining (C, left panel) and apoptosis was assessed by a caspase 3/7 reporter assay (C, right panel). Data are expressed as means from two independent experiments. Viability and cell amount values corresponding to CAM treatments are expressed as percentage relative to mock treatment set by default at 100%. Apoptosis values corresponding to CAM treatments as fold change relative to mock treatment set by default at 1. (D) *ANXA1* expression in HepAD38 cells expressing shRNA targeting *ANXA1* mRNA analysed by RT-qPCR. (E). Cells were either mock-treated or treated with either CAM-As or CAM-E and apoptosis levels were assessed by a caspase 3/7 reporter assay at Days 7 (left panel) and 14 (right panel) post-treatment. Data are expressed as means from two independent experiments. Apoptosis values corresponding to CAM treatments as fold change relative to mock treatment set by default at 1. Levels of significance: ∗*p* <0.05, ∗∗*p* <0.01, and ∗∗∗*p* <0.001 (two-tailed Mann–Whitney U test).

To understand the role of ANXA1 in the activation of apoptosis after HBc aggregation, we performed loss of function studies. First, we engineered HepG2-NTCP cells to express ANXA1-targeting shRNA leading to a marked knock-down of *ANXA1* expression compared with control cells (Fig. 7A). Cells expressing a non-targeting shRNA control underwent cell death after CAM-Ah RG7907 treatments, whereas the viability of cells expressing shRNA targeting *ANXA1* mRNA was much less affected (Fig. 7B-C). We also observed a tendency to lower levels of apoptotic cells upon ANXA1 knock-down compared with control shRNA expressing cells (Fig. 7C). However, longer CAM-A treatment induced loss of viability even after *ANXA1* knock-down (Fig. 7B, compare viability at Day 4 and Day 6). This observation suggests a role of ANXA1 in triggering apoptosis, although alternative pathways may eventually trigger cell death given the persistence of HBc aggregation in these cells, as highlighted by the transcriptomic dataset (Fig. 5). The impact of ANXA1 on cell viability was validated in a proof-of-principle assay using ANXA1-KO cells and the different CAM-A, further confirming the functional role of ANXA1 in inducing apoptosis although cells eventually die even in absence of ANXA1 after longer CAM-A treatment (Fig. S7). Finally, to reinforce the conclusions on ANXA1 involvement in triggering CAM-A induced apoptosis, we knocked down *ANXA1* expression in HepAD38 cells using RNAi (Fig. 7D) and monitored the activation of apoptosis. The decrease in *ANXA1* expression was associated with a reduction in the number of apoptotic cells after HBc aggregation as compared with the cells expressing a control shRNA (Fig. 7E). Taken together, our loss of function assay confirmed a key role of ANXA1 in the activation of apoptosis upon CAM-A dependent HBc aggregation in HBc-expressing and HBV-replicating cells.

## Discussion

Therapies targeting CHB with high functional cure rates are not yet available and represent an urgent unmet medical need. CAMs are considered appealing candidate drugs because of their strong antiviral properties against HBV.^6^ CAMs target the HBV core protein thus impairing capsid assembly, recycling, *de novo* infection and HBeAg production.^23^^,^[32], [33], [34], [35] Recently, we reported that CAM-A compounds induce a strong accumulation of HBc nuclear aggregates resulting in cell death both *in vivo* and *in vitro* models and are able to sustainably reduce circulating HBsAg in AAV-HBV mice models.^10^^,^^11^ Our results were further confirmed by an independent group using HBV infection models.^12^ In the present study, we extensively characterized this new mechanism of action specific to CAM-As. We first confirmed our previous observations in an *in vitro* HBV infection model. Of note, although we did detect cellular toxicity, we could not exclude at this stage that the decrease in HBsAg levels we observed may also have been attributable to a putative impact of CAM-A treatment on multiple HBV replication steps. Moreover, given the low infection rates and given the hypothesis that high levels of core are necessary to induce CCD,^12^ we developed alternative models to characterize this secondary mode of action. We demonstrated that all CAM-A compounds induce apoptosis through the accumulation of nuclear HBc aggregates. Indeed, CAM-A treatments did not affect the viability of cells expressing HBc CAM-resistant mutant T33N, ruling out the possibility that apoptosis is a result of an off-target effect of CAM-A compounds. Interestingly, the I105F and P25A mutations in the CAM-binding pocket found in HBV clinical isolates^25^^,^^36^^,^^37^ are still sensitive to CAM-A treatments although with a lower efficacy. These results demonstrate the importance of improving CAM-A structure to make them more efficient against resistant mutations that might appear and also suggest that using CAMs in combination therapy with NUCs would be more effective in suppressing the emergence of CAM-resistant mutants.

Moreover, we demonstrated that CAM-A treatment also induces apoptosis in HBc-expressing PHH, confirming that this phenotype is not the result of disrupted pathways specific of the liver-derived cancer cell lines but is specifically caused by the accumulation of HBc aggregation into the nucleus.

RNA sequencing provided us with a candidate list of deregulated genes after CAM-A-dependent HBc aggregation. As expected, we identified several proteins that are known to participate in the activation of apoptosis, and we focused our attention on ANXA1, given its central role in the induction of apoptosis. ANXA1 belongs to the Annexin family of proteins.^27^ ANXA1 is known for its involvement in the anti-inflammatory response, apoptosis, and viral infection.[29], [30], [31] Interestingly, ANXA1 can also promote apoptosis of lung epithelial cells infected by influenza A virus through RIG-I signaling.^31^ Our findings also showed that the upregulation of ANXA1 expression was HBV-dependent and only occurred after HBc aggregation. Of note, apoptosis is not fully abolished in absence of ANXA1 suggesting that other factors contribute to this phenotype. The next step will be to investigate the role of other proteins that have been identified in our RNA seq analysis. In the future, it would be also interesting to study ANXA1 expression in CAM-A treated patients.

CAM-A compounds abrogate the ability of HBc to form mature capsid structures and to internalize the polymerase-pgRNA complex, hence leaving free pgRNA molecules in the cytoplasm that can be sensed by the RNA sensor RIG-I. Indeed, RIG-I can bind the pgRNA at the level of the ε−structures.^38^ These observations raise the question of whether CAM molecules can induce an innate immune response against HBV. Interestingly, in our previous study using an AAV-HBV mouse model we found that CAM-A treatment, but not CAM-E, induced an innate immune response prominently featuring the ISG15 pathway.^10^ Further observations are needed to make the link between pgRNA, innate immune response and CCD in more relevant models.

Clinically this mechanism of action may contribute to the antiviral effect by specifically turning over infected hepatocytes. CCD also encourages the proliferation of uninfected hepatocytes while cccDNA is lost during mitosis. The gradual reduction of infected hepatocytes might promote the immune control of the infection or elimination of infection altogether. In this context, we observed CCD in HBV-infected dHepaRG cells treated with RG7907 at 10 μM, equivalent to about 40 times its EC90 value for the primary mechanism of action. It still remains to be determined whether the concentrations used in patients align with CCD phenotype.

Taken together, we elucidated a novel mechanism of action by which CAM-A affects the replication cycle of HBV by inducing apoptosis of core-expressing cells. Our results pave the way for a better understanding of the CAM-A mod of action and open the door to new therapeutic strategies based on CAM treatment to decrease HBsAg levels in patients with chronic HBV infections.

## Abbreviations

CAMs, Capsid assembly modulators; cccDNA, covalently closed circular DNA (cccDNA); DMSO, dimethyl sulfoxide; HBc, HBV core antigen; CC, hepatocellular carcinoma; IV, human immunodeficiency virus; LDH, lactate dehydrogenase; NEAA, non-essential amino acids; NUCs, nucleos(t)ide analogues; pgRNA, pre-genomic RNA; PHH: primary human hepatocytes; sgRNA, Small guide RNA; VSV-G, vesicular stomatitis virus glycoprotein; Vge, viral genome equivalents.

## Financial support

This work of the Interdisciplinary Thematic Institute IMCBio, as part of the ITI 2021-2028 program of the 10.13039/501100003768University of Strasbourg, CNRS and 10.13039/501100001677Inserm, was supported by IdEx Unistra (ANR-10-IDEX-0002), and by SFRI-STRAT’US project (ANR-20-SFRI-0012) and 10.13039/501100001828EUR IMCBio (ANR-17-EURE-0023) under the framework of the French Investments for the Future Program. T. F. B and E. R. V. received funding from Aligos Belgium BV as part of the 10.13039/100012331VLAIO project CoHeBA (HBC.2020.2454). V.T. et E.R.V. acknowledges fundings from 10.13039/501100003323ANRS - Maladies infectieuses émergentes - (ANRS-MIE, grant number ANRS0543). E.R.V. acknowledges fundings from the 10.13039/501100001665French National Research Agency (ANR, grant number ANR-21-CE15-0035-01 DELTArget). T.F.B acknowledges funding from the European Union (EUERC-AdG-2014-HEPCIR #671231) and 10.13039/501100004097ARC Foundation TheraHCC2.0 (IHU201901299).

## Conflicts of interest

T. F. B and E. R. V. received funding from Aligos Belgium BV as part of the VLAIO project CoHeBA (HBC.2020.2454) with V. T.’s fellowship funded by the grant. Y. D., D. B. K. and H. V. are employees of Aligos and may own stock.

Please refer to the accompanying ICMJE disclosure forms for further details.

## Authors’ contributions

Study concept and design: T.F.B., Y.D., and E.R.V. Study supervision: V.T. and E.R.V. Acquisition of data: V.T. with the support of L.M-H and C.G. Analysis and interpretation of data: C.G. V.T., L.M-H, C.G., H.V., D.B.K., C.S., T.F.B., Y.D., and E.R.V. Administrative, technical, or material support: F.G. and P.P.

Drafting of the manuscript V.T. and E.R.V. All the authors approved the manuscript.

## Data availability statement

The original data from this study are available through the corresponding author upon reasonable request. Full Western blot figures are provided in Supplementary Figs. S8 to S11. The RNAseq raw data are available through the Gene Expression Omnibus data repository (GSE263292): https://www.ncbi.nlm.nih.gov/geo/query/acc.cgi?acc=GSE263292.
