# Supplementary material for: CAM-A-dependent HBV core aggregation induces apoptosis through ANXA1
Source: JHEP Rep. 2024 Jun 10;6(10):101134. doi: 10.1016/j.jhepr.2024.101134 (PMC11462251; doi:10.1016/j.jhepr.2024.101134)
Supplement: Multimedia Component 1 [file mmc1.pdf]

CAM-A-dependent HBV core aggregation induces apoptosis through ANXA1

Valerio Taverniti, Laura Meiss-Heydmann, Cloé Gadenne, Hannah Vanrusselt, Dieudonné Buh Kum, Fabio Giannone, Patrick Pessaux, Catherine Schuster, Thomas F. Baumert, Yannick Debing, Eloi R. Verrier

Table of contents

Fig. S1……2

Fig. S2……3

Fig. S3……3

Fig. S4……4

Fig. S5……4

Fig. S6……5

Fig. S7……5

Fig. S8……6

Fig. S9……6

Fig. S10……7

Fig. S11……7

Supplementary Figures

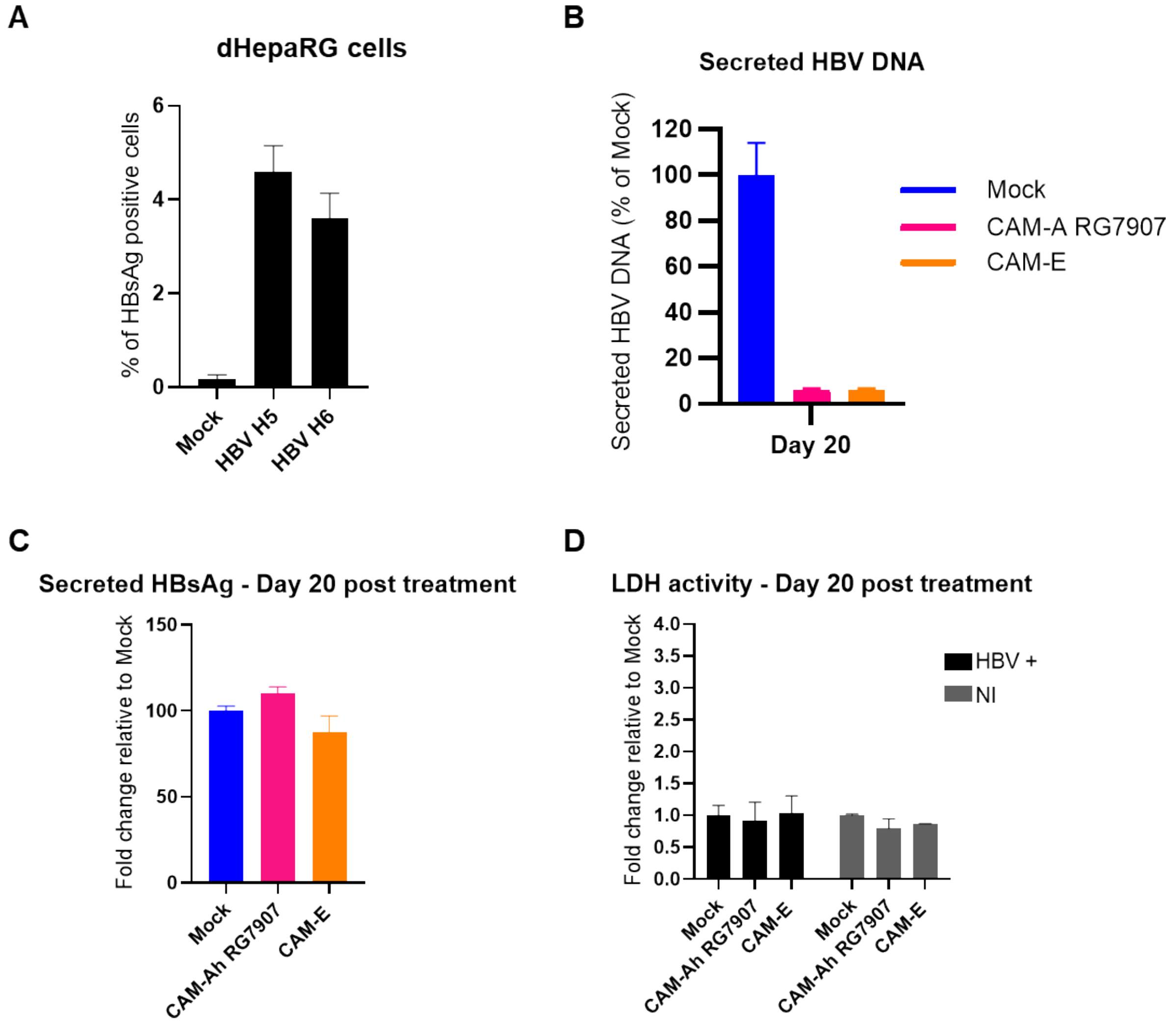

Fig. S1. Short term CAM-Ah RG7007 treatment does not alter HBsAg secretion and cell viability. (A) HBV-infection levels in the two independent assays presented in Figure 1. (B-C) The efficiency of CAM treatment was assessed by measuring secreted HBV DNA (B) and secreted HBsAg (C) from one proof-of-principle assay. (C) Cytotoxicity was quantified by measuring the LDH activity in the cell supernatant of treated cells. Values are normalized to mock treated cells set by default at 1.

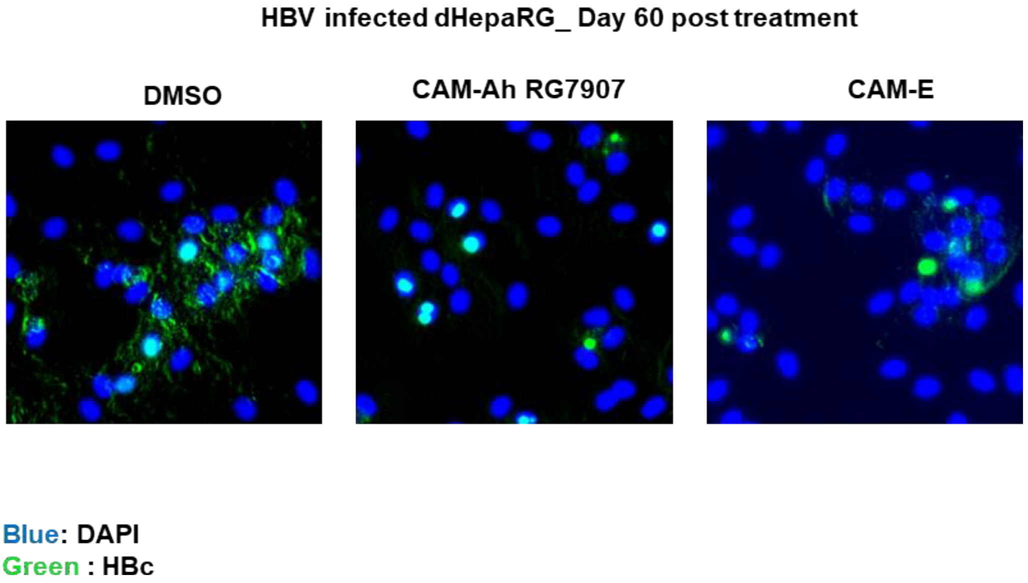

Fig. S2. CAM-A treatment induce HBc aggregates accumulation in the nucleus of HBV infected dHepaRG. Immunofluorescent (IF) staining for HBc (green) and DAPI-stained nuclei (blue) in HBV infected dHepaRG treated with DMSO, CAM-Ah RG7907 and CAM-E for 60 days.

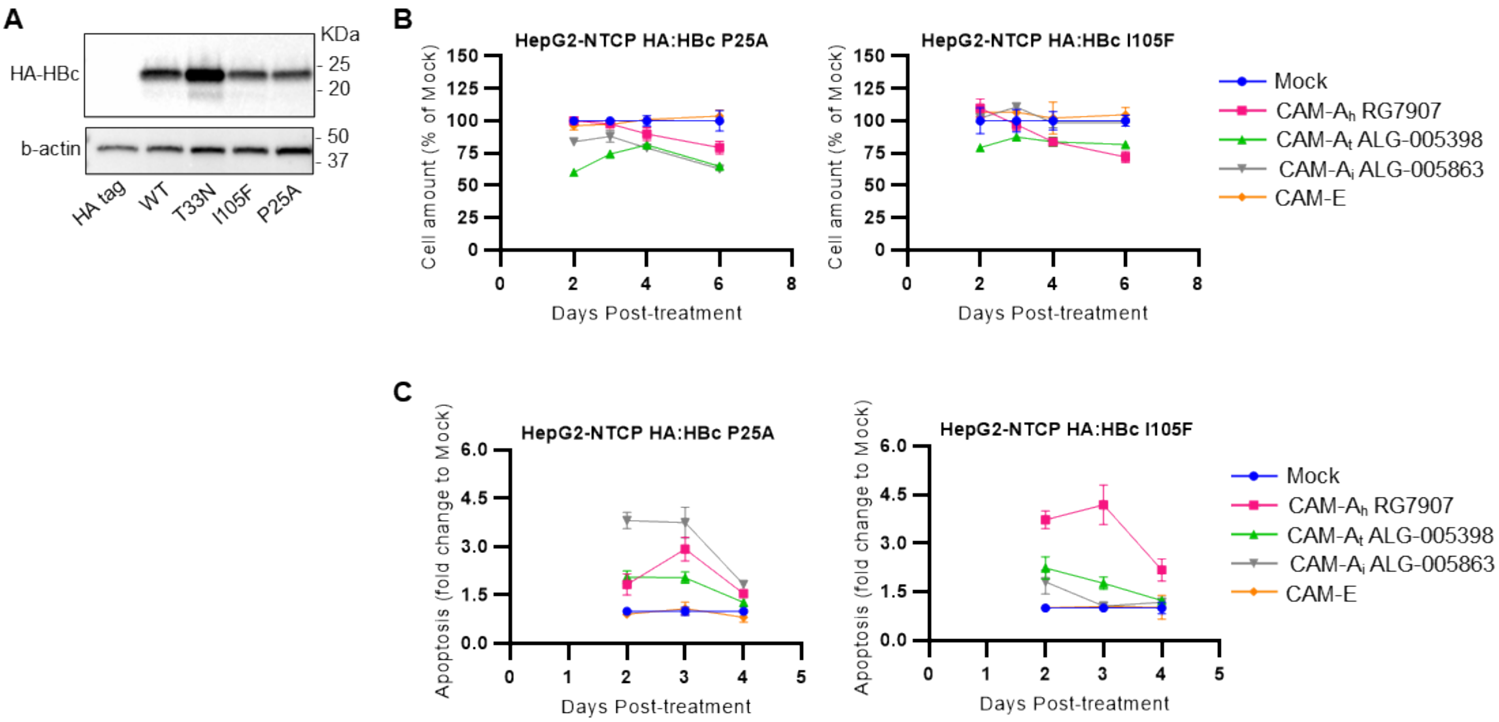

Fig. S3. CAM-As induced apoptosis in HBc WT-overexpressing cells. (A) Western blot of HA:HBc WT and HA:HBc T33N or I105F or P25A mutants-overexpressing HepG2-NTCP cell lysates probed for HA. (B-C) Cells were either mock-treated with 2% DMSO or treated with either CAM-A$_h$ RG7907 or CAM-A$_t$ ALG-005398 or CAM-A$_i$ ALG-005863 or CAM-E (final concentration 1 μM) for the indicated time and total cell number was assessed by DAPI staining (B), and apoptosis levels were assessed by a caspase 3/7 reporter assay (C).

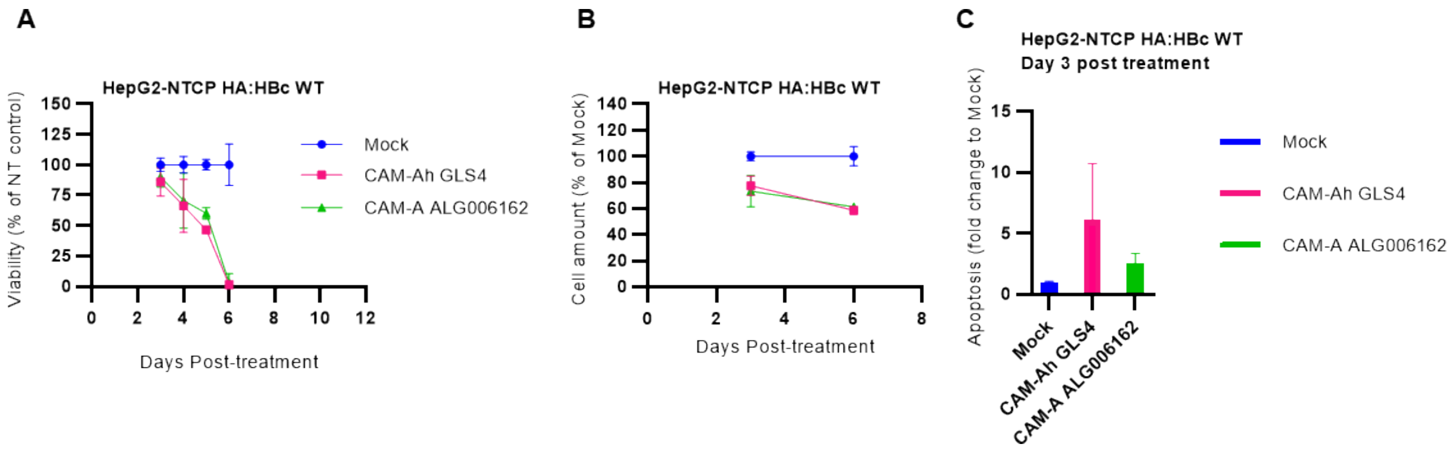

Fig. S4. Effects of CAM-A$_h$ and CAM-A$_t$ on cell viability and apoptosis. HA:HBc WT overexpressing HepG2-NTCP were either mock-treated with 2% DMSO or treated with either CAM-A$_h$ GLS4 or CAM-A$_t$ ALG-006162 and assessed for cell viability by PrestoBlue, total cell amount by DAPI staining, and apoptosis by a caspase 3/7 reporter assay.

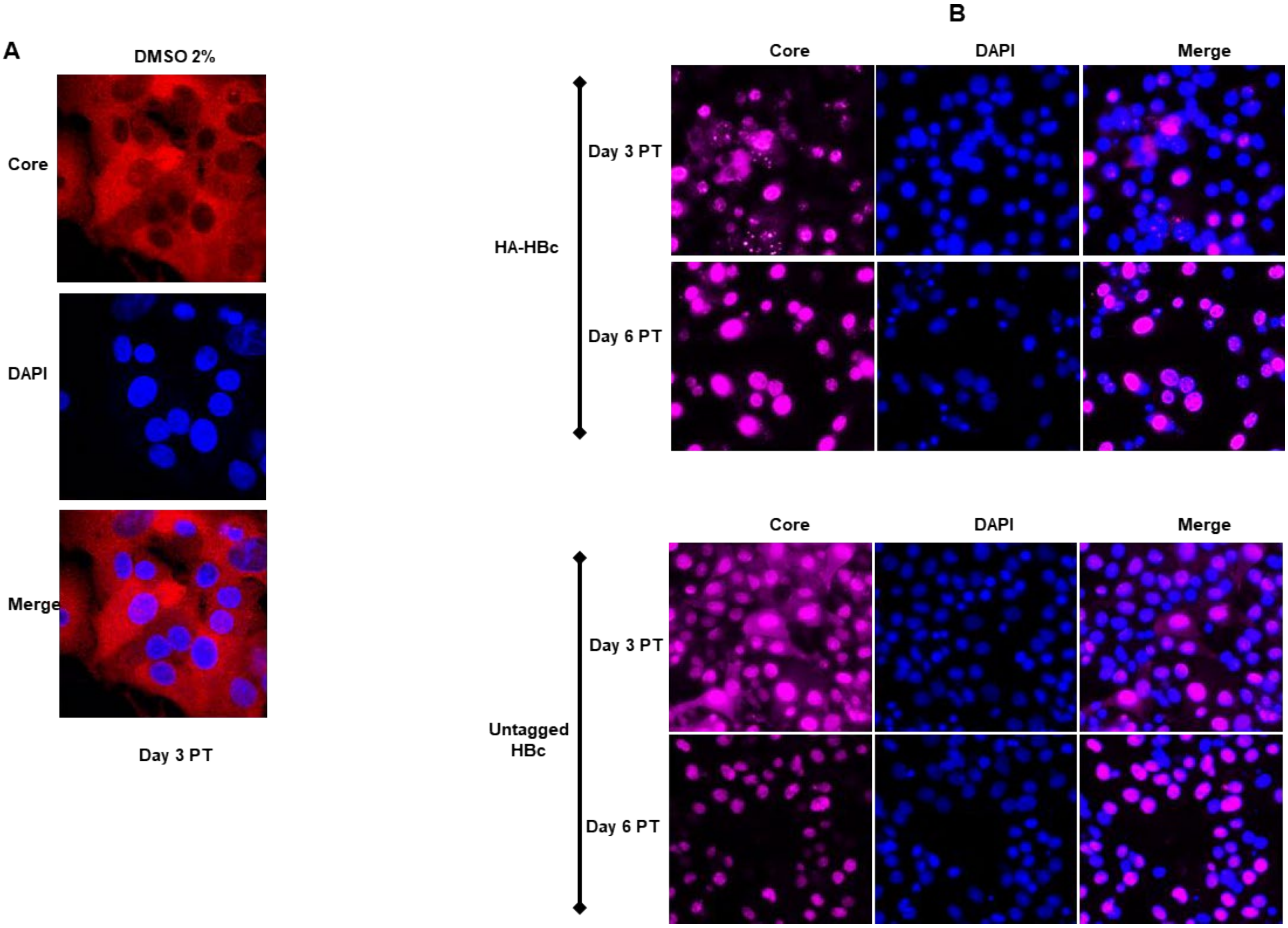

Fig. S5. HBc cellular localization. (A) Immunofluorescent (IF) staining for HBc (red) and DAPI-stained nuclei (blue) in HA:HBc treated with DMSO for 3 days. (B) Immunofluorescent (IF) staining for HBc (red) and DAPI-stained nuclei (blue) in HA:HBc or untagged HBc treated with DMSO for 3 days and 6 days.

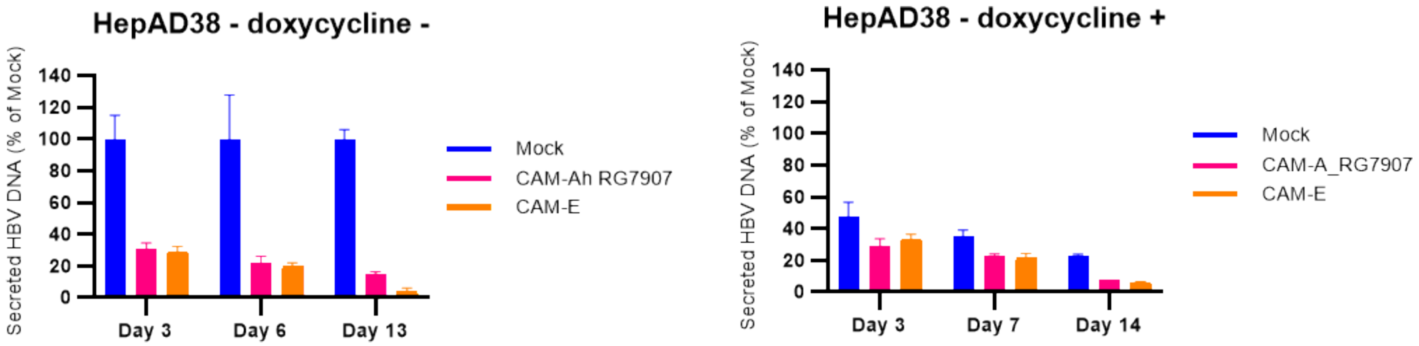

Fig. S6. Secreted HBV particles in HepAD38 cultured in presence of doxycycline. HepAD38 cells were either mock-treated with 2% DMSO or treated with either CAM-A$_h$ RG7907 or CAM-E (final concentration 1 µM) in presence or absence of doxycycline. Viral loads were assessed by quantifying the amount of secreted HBV DNA by qPCR. One proof-of-principle assay with three biological replicates is shown.

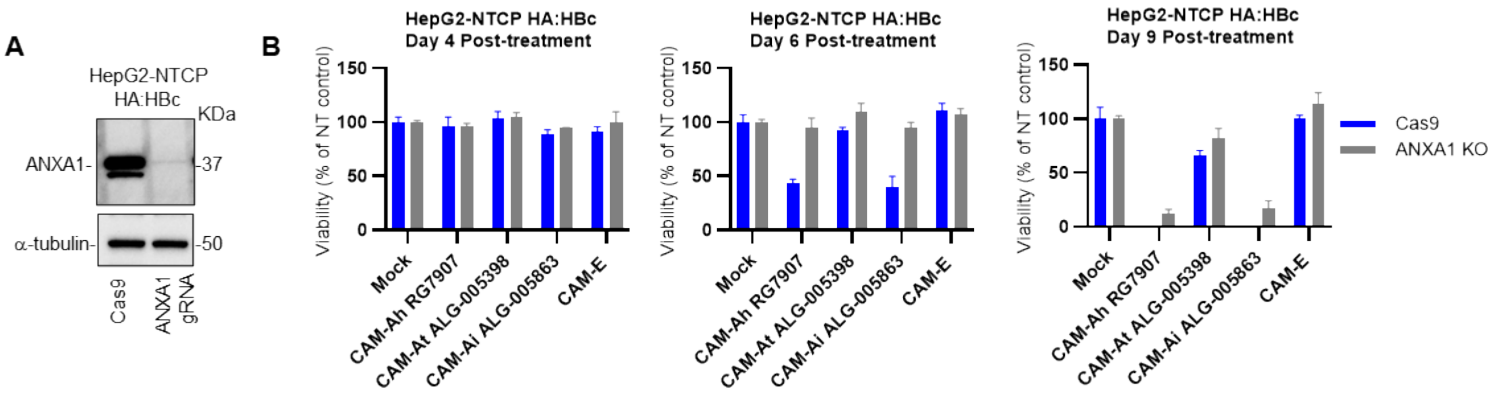

Fig. S7. ANXA1 KO is associated with delayed apoptosis. (A) ANXA1 Knock out measured by Western blot in HepG2-NTCP HA:HBc expressing the Cas9 or a gRNA targeting ANXA1. (B) Cells were either mock-treated or treated with either CAM-As or CAM-E for the indicated time and assessed for cell viability by PrestoBlue. (C) ANXA1 expression in HepAD38 expressing shRNA targeting ANXA1 analyzed by RT-qPCR (D) Cells were either mock-treated or treated with either CAM-As or CAM-E and assessed for apoptosis by a caspase 3/7 reporter assay at days 7 and 14 post-treatment. Data on HepG2-NTCP HA:HBc result from two independent experiments performed in triplicate (N=6). HepAD38 data result from an experiment performed in triplicate. Viability values corresponding to CAM treatments are expressed as percentage relative to mock treatment set by default at 100%. Apoptosis values corresponding to CAM treatments as fold change relative to mock treatment set by default at 1.

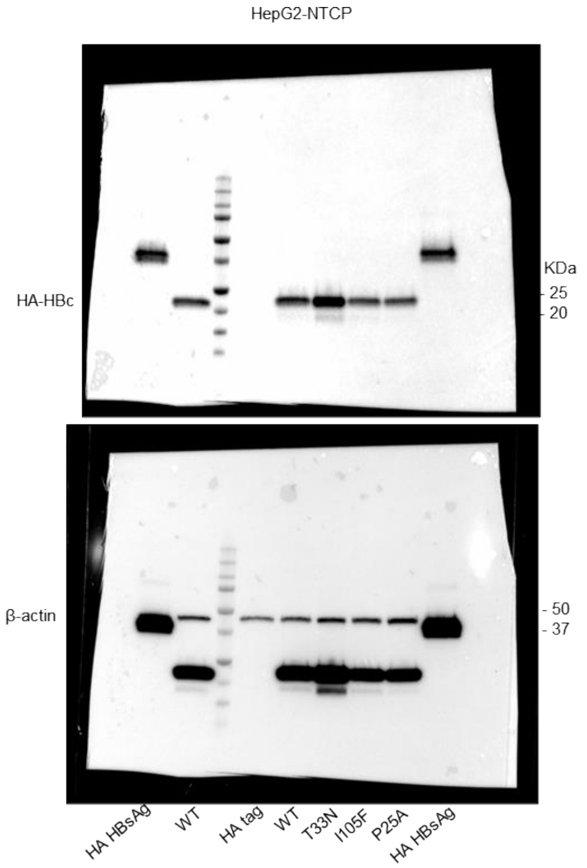

Fig. S8. Original Western blot figures related to Figure 2A and Fig. S3A.

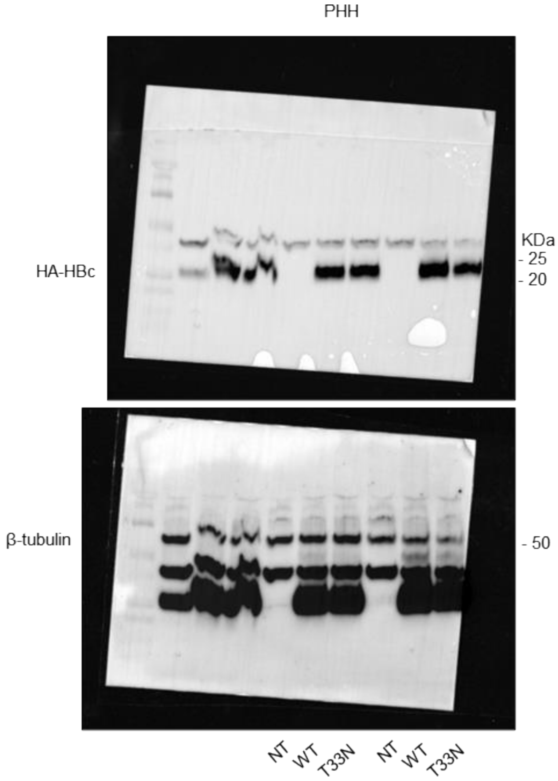

Fig. S9. Original Western blot figures related to Figure 4B.

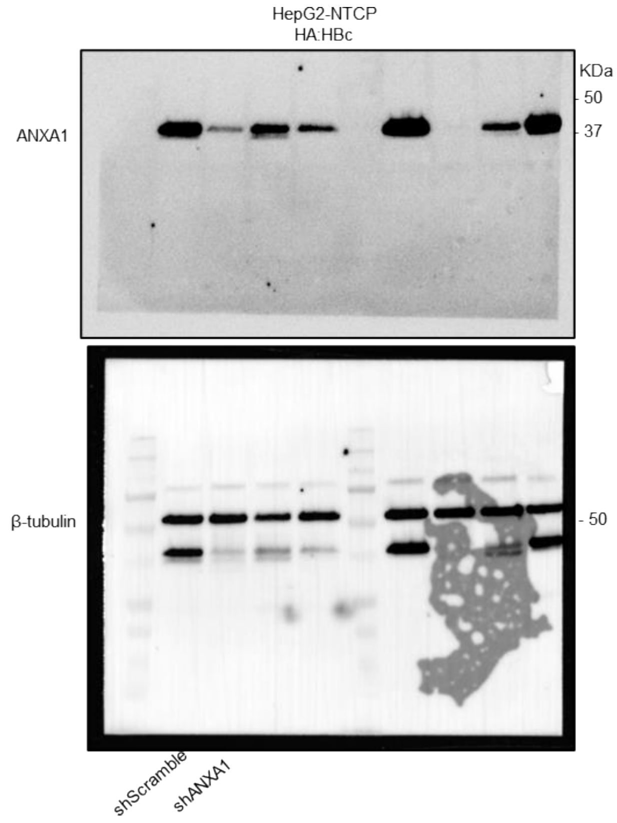

Fig. S10. Original Western blot figures related to Figure 7A.

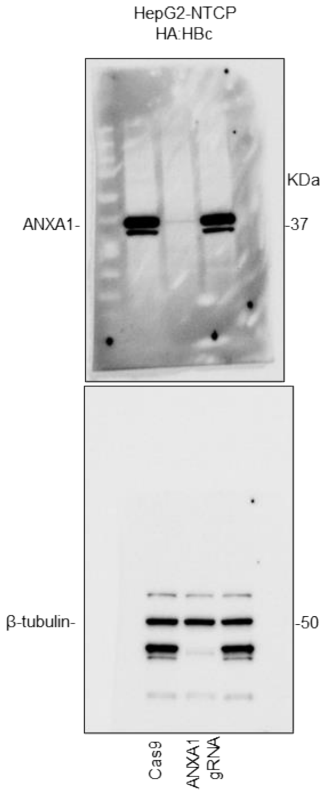

Fig. S11. Original Western blot figures related to Fig. S7A.
