# Supplementary material for: CAM-A-dependent HBV core aggregation induces apoptosis through ANXA1
Source: JHEP Rep. 2024 Jun 10;6(10):101134. doi: 10.1016/j.jhepr.2024.101134 (PMC11462251; doi:10.1016/j.jhepr.2024.101134)
Supplement: Multimedia Component 2 [file mmc2.pdf]

JOURNAL
OF HEPATOLOGY

# Journal of Hepatology

## CTAT methods

Tables for a "Complete, Transparent, Accurate and Timely account" (CTAT) are now mandatory for all revised submissions. The aim is to enhance the reproducibility of methods.

- Only include the parts relevant to your study
- Refer to the CTAT in the main text as 'Supplementary CTAT Table'
- Do not add subheadings
- Add as many rows as needed to include all information
- Only include one item per row

**If the CTAT form is not relevant to your study, please outline the reasons why:**

### 1.1 Antibodies

| Name | Citation | Supplier | Cat no. | Clone no. |
|---|---|---|---|---|
| Anti-HA tag antibody | | Abcam | ab9110 | Rabbit Polyclonal |
| Anti-Hepatitis B Virus Core Antigen antibody | | Abcam | ab8637 | C1 |
| Goat anti-Mouse IgG (H+L) Secondary Antibody, Alexa Fluor™ Plus 488 | | Invitrogen | A32723 | Polyclonal |
| Goat anti-Rabbit IgG Secondary Antibody, Alexa Fluor™ 647 | | Invitrogen | A-21245 | Polyclonal |
| Anti-HA High Affinity | | Sigma | 11867423001 | 3F10 |
| Recombinant Anti-Annexin A1/ANXA1 antibody | | Abcam | Ab214486 | EPR19342 |
| Anti-beta Actin antibody | | Abcam | AB8226 | mAbcam 8226 |
| beta Tubulin antibody | | Genetex | GTX101279 | Polyclonal |

## 1.2 Cell lines

| Name | Citation | Supplier | Cat no. | Passage no. | Authentication test method |
|---|---|---|---|---|---|
| HepaRG | Gripon et al., PNAS 2002 doi:10.1073/pnas.23213769 | Biopredic | HPR101 | Below 20 | None |
| HepG2-NTCP | Eller, Heydmann et al., Nat Commun 2020 doi: 10.1038/s41467-020-16517-w | Homemade | NA | Below 20 | None |
| HEK 293T | | ATCC | CRL-3216 | Below 20 | None |
| HepAD38 | Ladner et al., Antimicrob Agents Chemother 1997 | E. Hildt, Paul-Ehrlich-Institut, Langen, GER. | NA | Below 20 | None |

## 1.3 Organisms

| Name | Citation | Supplier | Strain | Sex | Age | Overall n number |
|---|---|---|---|---|---|---|
| | | | | | | |

## 1.4 Sequence based reagents

| Name | Sequence | Supplier |
|---|---|---|
| pc/pgRNA Fw primer | GGTCCCCTAGAAGAAGAACTCCCT | Merck |
| pc/pgRNA Re primer | CATTGAGATTCCCGAGATTGAGAT | Merck |
| pc/pgRNA probe | TCTCAATCGCCGCGTCGCAGA-[BHQ1] | Merck |
| ANXA1 TaqMan assay | Proprietary | Applied Biosystems ID: Hs00167549_m1 |
| GAPDH TaqMan assay | Proprietary | Applied Biosystems ID: Hs02786624_g1 |
| HBV DNA (BC1) | GGAAAGAAGTCAGAAGGCAA | Merck |
| HBV DNA (PGP) | CACCTCTGCCTAATCATC | Merck |

## 1.5 Biological samples

| Description | Source | Identifier |
|---|---|---|
| Primary Human Hepatocytes isolated from liver resections | Strasbourg University Hospitals, Strasbourg, France | Ethics Committee of the Strasbourg University Hospitals (CPP) and the Ministry of Higher Education and Research of France (DC 2016 2616) |

## 1.6 Deposited data

| Name of repository | Identifier | Link |
|---|---|---|
| GEO | *Pending* | *Pending* |

## 1.7 Software

| Software name | Manufacturer | Version |
|---|---|---|
| GraphPad Prism | GraphPad Software | 9 |
| Image lab | Bio-Rad | 6.1 |
| QuantStudio TM Real time PCR software | Applied Biosystem | 1.5.1 |
| ZEN | Carl Zeiss | 2.3 |

## 1.8 Other (e.g. drugs, proteins, vectors etc.)

| | | |
|---|---|---|
| CAM-Ah RG7907 | Aligos therapeutics | |
| CAM-At ALG005398 | Aligos therapeutics | |
| CAM-Ai ALG005863 | Aligos therapeutics | |
| CAM-Ah GLS4 | Aligos therapeutics | |
| CAM-At ALG006162 | Aligos therapeutics | |
| CAM-E | Aligos therapeutics | |
| pLenti- HA-tagged HBc WT | Vectorbuilder | VB210422-1136fgt |
| pLenti- HA-tagged HBc T33N | Vectorbuilder | VB220105-1043neq |
| pLenti- HA-tagged HBc P25A | Vectorbuilder | VB220105-1041sqy |
| pLenti- HA-tagged HBc I105F | Vectorbuilder | VB220105-1046gga |
| pLenti- HA control | Vectorbuilder | VB220105-1047unq |
| pLenti shRNA *ANXA1* | Vectorbuilder | VB900130-7607sfz |
| pLenti shRNA non target | Vectorbuilder | VB010000-0005mme |
| pLenti ANXA1 gRNA | Genescript | SC1805 |

## 1.9 Please provide the details of the corresponding methods author for the manuscript:

Eloi R. Verrier, PhD
Inserm U1110
3 rue Koeberlé
67000 Strasbourg
France
e.verrier@unistra.fr
+33368853706

**2.0 Please confirm for randomised controlled trials all versions of the clinical protocol are included in the submission. These will be published online as supplementary information.**

| |
|---|
| NA |
